# Utilization of Corncob as an Immobilization Matrix for a Xylanolytic Yeast Strain

**DOI:** 10.3390/polym15030683

**Published:** 2023-01-29

**Authors:** Maham Aftab, Uroosa Ejaz, Rami Adel Pashameah, Aimen Fatima, Jaweria Syed, Immad Ansari, Muhammad Sohail, Samah A. AlSubhi, Eman Alzahrani, Zeinhom M. El-Bahy

**Affiliations:** 1 Department of Biosciences, Faculty of Life Sciences, Shaheed Zulfikar Ali Bhutto Institute of Science and Technology (SZABIST), Karachi 75600, Pakistan; 2 Department of Chemistry, Faculty of Applied Science, Umm Al-Qura University, Makkah 24230, Saudi Arabia; 3 Department of Microbiology, University of Karachi, Karachi 75270, Pakistan; 4 Laboratory Medicine Department, Faculty of Applied Medical Science, Umm Al-Qura University, Makkah 24230, Saudi Arabia; 5 Department of Chemistry, College of Science, Taif University, P.O. Box 11099, Taif 21944, Saudi Arabia; 6 Department of Chemistry, Faculty of Science, Al-Azhar University, Nasr City 11884, Egypt

**Keywords:** alkali pretreatment, corncob, immobilization, xylanase, yeast

## Abstract

Immobilization of microbial cells for the production of industrially important enzymes has been reported to offer the advantages of recyclability, higher yields and cost effectiveness. The search for an appropriate matrix that is affordable and easy to prepare is a significant topic in microbial biotechnology. Here, an abundant type of agro-industrial waste—corncob—was utilized as an immobilization matrix for the production of xylanase from an indigenous yeast strain, *Saccharomyces cerevisiae* MK-157. This is the first report describing xylanase production from immobilized *S. cerevisiae*. To render the corncob matrix more porous, alkaline pretreatment was undertaken and yeast cells were immobilized on the matrix by cultivating at 30 °C for 48 h in Sabouraud dextrose broth. After incubation, the immobilized matrix was transferred to mineral salt medium containing 1% xylan and incubated at 30 °C for 24 h. Xylanase production was determined in cell-free culture supernatant and the matrix was recycled for up to seven cycles. Moreover, xylanase-mediated saccharification was carried out using sugarcane bagasse as a substrate and the release of reducing sugars was monitored. The results showed that the immobilized yeast produced 4.97 IU mL^−1^ xylanase in the first production cycle, indicating a >tenfold increase compared to the free cells. Xylanase production further increased to its maximum levels (9.23 IU mL^−1^) in the fourth production cycle. Nonetheless, the cells retained 100% productivity for up to seven cycles. The volumetric and specific productivity of xylanase were also the highest in the fourth cycle. Scanning electron microscopy images revealed the rough surface of the untreated corncob, which became more porous after alkaline pretreatment. Immobilized yeast cells were also visible on the corncob pieces. The saccharification of a natural resource—sugarcane bagasse—using xylanase preparation yielded 26 mg L^−1^ of reducing sugars. Therefore, it can be concluded that yeast strains can yield sufficient quantities of xylanase, allowing possible biotechnological applications. Moreover, corncob can serve as a cost-effective matrix for industrially important yeast strains.

## 1. Introduction

Xylan, a constituent of plant cell walls, is regarded as the most abundant, renewable polysaccharide after cellulose. It is a heteropolysaccharide consisting of different sugars, such as D-mannose, D-galactose and L-arabinose, and organic acids, such as glucuronic acid, ferulic acid and acetic acid, interwoven together with the help of ester and glycosidic bonds. The breakdown of xylan is hindered due to its heterogeneous nature; however, xylanases comprise a group of enzymes capable of cleaving the heterogeneous β-1,4-glycoside linkage. Xylanase is an industrially important enzyme that is used in various sectors, such as the paper and pulp, animal feed processing, biofuel and brewing industries. Over the past few years, the market share of xylanase has increased significantly [1,2]. The cost of enzyme production is the main impediment in exploring new fields for the application of the enzyme. Therefore, the demand for industrial enzymes of microbial origin is increasing rapidly due to their cost-effective production. The diversity associated with microbial species and the nature of the habitats from which these organisms are isolated are reflected in the properties of their metabolites [3]; hence, microorganisms appear to be a suitable source of industrially relevant enzymes, including xylanases [4]. Indeed, tolerance to a wide range of temperatures and pH levels renders microbial xylanase an ideal candidate for work in different industries [5].

*Saccharomyces cerevisiae* has long been known for its ability to produce alcohol and for its application in bread making. The ability to utilize glucose under aerobic and anaerobic conditions with a shorter generation time than molds makes this yeast an ideal microbial cell factory [6]. Furthermore, the tolerance of this yeast to acidic environments and high sugar levels also provides it with advantages over bacterial cell factories. The well-characterized genome of this yeast has been exploited to allow its use as an ideal eukaryotic host for heterologous protein expression [7]. However, the enzymatic potential of *S. cerevisiae* is not widely recognized; hence, there are few reports on the production of industrial enzymes using this yeast. The MK-157 strain of *S. cerevisiae* has been previously highlighted for the production of pectinase [8] and cellulase [9]. However, its xylanolytic potential has not been investigated yet.

The production cost of industrial enzymes is the main impediment in their widespread application. Immobilization is well-recognized for its effectiveness as a cost-cutting strategy and for the development of continuous immobilized cell reactors. Moreover, immobilization improves the stability of microbial cells under extreme pH and temperature conditions and aids in developing continuous processes [10]. However, the selection of the support material is extremely important in an immobilization process, as the matrix influences the viability of the immobilized organisms. The carriers must be insoluble, non-biodegradable, non-toxic, reasonably priced and easy to handle and regenerate. Furthermore, the matrix should have the capacity to hold excessive microbial mass with high mechanical and chemical stability and should not hinder nutrient diffusion to its core [11]. The ideal matrix is difficult to find, but corncobs possess many of these desirable attributes. Being an agricultural waste product, it is inexpensive and abundantly available in many parts of the world. Its porous structure can entrap microbial cells, and it is not degraded in most microbiological media. It has high surface area, with microtubules that provide safe habitats for microbial cells [12]. Although we have previously described the utilization of corncobs as an immobilization matrix [8], this work holds significance as it reports the xylanolytic potential of a yeast that has not been previously investigated for this activity. An improvement in xylanase production was achieved here.

Furthermore, advances in the technology used to convert lignocellulosic biomass to biofuel are impeded by the production costs of the enzymes. Sugarcane bagasse is an abundant lignocellulose resource that can yield various value-added products upon saccharification. Global annual production of sugarcane (*Saccharum officinarum*) amounts to about 1.6 billion tons. It is squeezed to obtain juice, resulting in concomitant production of 279 million metric tons of the byproduct bagasse [13]. The global outlook for sugarcane production shows that Brazil is currently the largest producer at about 739,300 metric tons per year, followed by India, China, Thailand and Pakistan [14]. Bagasse is a feedstock that can potentially be used in numerous applications due to its chemical composition, which includes lignin (21%), hemicellulose (28%), cellulose (44%), ash (5%) and extractive components (2%) [15]. Saccharification is an essential step when transforming bagasse into high-quality fermentable sugars [16]. Saccharification of lignocellulosics can be achieved using acids or alkalis, but enzymatic processes require mild process conditions and, hence, are preferred over chemical hydrolysis [17]. However, the cost of enzymes can limit the development of enzyme-mediated saccharification; therefore, production of enzymes through immobilized cells using cost-effective materials is a promising strategy.

In this study, *S. cerevisiae* MK-157 was immobilized on corncob and xylanase production using immobilized yeast was evaluated. The immobilized matrix was recycled to check the efficiency of the immobilized cells. The xylanase was evaluated for its efficiency in saccharifying sugarcane bagasse.

## 2. Materials and Methods

### 2.1. Inoculum Preparation

The yeast strain *S. cerevisiae* MK-157 (accession number: KY748281.1) was obtained from the culture collection of the Department of Microbiology, University of Karachi, Pakistan. The culture was revived on Sabouraud dextrose agar (SDA) and incubated at 30 °C for 48 h. To prepare the inoculum, an isolated colony of *S. cerevisiae* MK-157 was inoculated in SDB and incubated at 30 °C for 48 h. The OD_600_ of the culture was maintained at 1.0.

### 2.2. Effect of Media Composition on Xylanase Production

Yeast extract peptone (YEP) medium, peptone broth medium (PBM) medium [18] and mineral salt medium (MSM) [9] supplemented with 1% (*w*/*v*) beechwood xylan were used to check the effect of media on xylanase production. A 10% (*v*/*v*) inoculum was added into the production media, and all the samples were incubated at 30 °C for 24 h. Post-incubation media were centrifuged at 7000 rpm for 15 min and supernatant was collected to perform the xylanase assay.

### 2.3. Xylanase Assay

To assess xylanase activity, 25 μL of cell-free culture supernatant was mixed with 25 μL of substrate. The substrate was prepared by producing 0.5% (*w*/*v*) beechwood xylan solution in 50 mM sodium citrate buffer at pH 4.8. The mixture of enzyme and substrate was incubated at 30 °C for 15 min. After the reaction time, 150 μL of dinitrosalicylic acid (DNS) reagent was added and the mixture was boiled for 5 min. Distilled water (720 μL) was added and absorbance at 540 nm was recorded. The reaction using heat-inactivated enzyme was used as a blank. For the enzyme activity, one international unit (IU) was defined as the amount of enzyme required to release 1 µM of xylose per ml per min under standard assay conditions [19].

### 2.4. Pretreatment of Corncob

**Figure 1:**
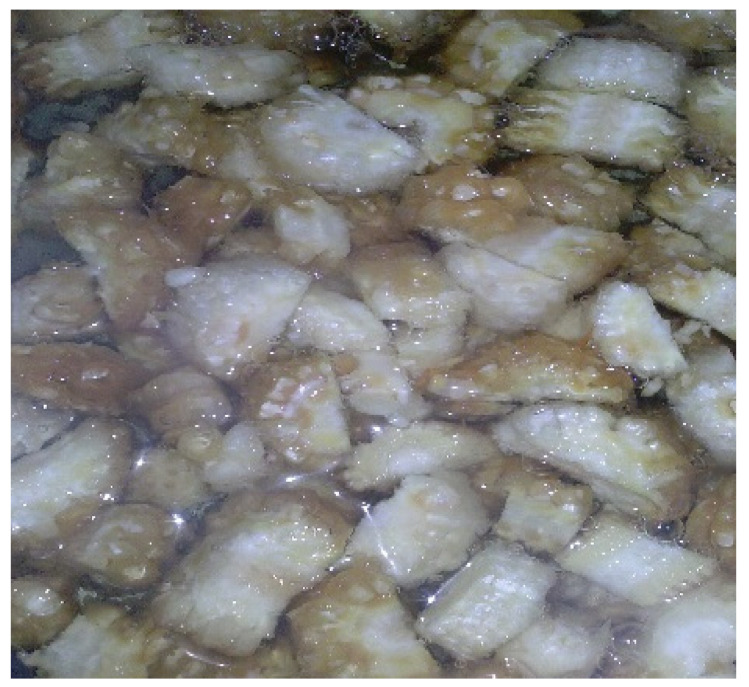
Image of boiled corncob surface.

To prepare the matrix, corncobs were procured locally, washed and dried. They were cut into pieces of 0.5 ± 0.05 cm^3^ size, washed with water and boiled for 5 min (Figure 1). These pieces were then dried at 50 °C for 48 h. For pretreatment, corncob pieces were loaded in 1% sodium hydroxide at a rate of 50 mL g^−1^ and kept at 30 °C for 24 h. The pieces were washed with tap water until the pH of the matrix and filtrate became neutral. Afterwards, alkali-pretreated corncob pieces were dried in an oven for 48 h at 50 °C.

### 2.5. Immobilization on Corncob

A 10% (*v*/*v*) inoculum was transferred to 25 mL SDB containing two pieces of alkali-treated corncob and incubated at 30 °C for 48 h. The weight of the matrix before and after the experiment was also noted.

### 2.6. Xylanase Production from Immobilized Yeast

**Figure 2:**
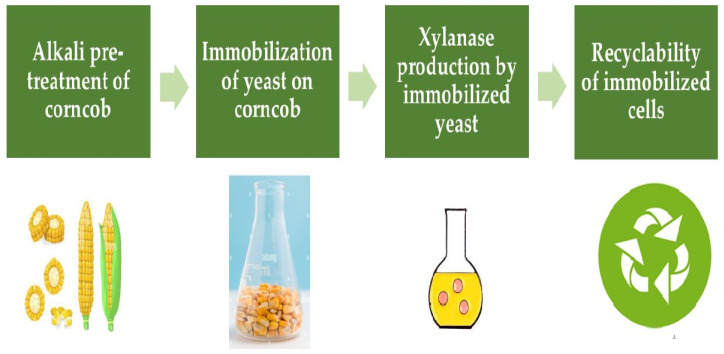
Flow chart showing xylanase production from immobilized yeast.

For the production of xylanase from immobilized yeast, two pieces of immobilized matrix were transferred into the flasks containing 25 mL MSM supplemented with 1% (*w*/*v*) beechwood xylan as a substrate. To draw a comparison, free cells were also inoculated separately from the flasks containing the same medium. The flasks were incubated at 30 °C for 24 h. Post-incubation media was centrifuged at 7000 rpm for 15 min and supernatant was collected to perform the xylanase assay, whereas the immobilized matrix was aseptically transferred to the fresh MSM for the second cycle. A similar process was repeated for up to eight cycles. The specific productivity per g of the substrate (xylan) and the volumetric productivity (IU mL^−1^ h^−1^) were calculated for different production cycles to compare the enzyme yields. A flow chart of the methodology is presented in Figure 2.

### 2.7. Scanning Electron Microscopy

Untreated, alkali-pretreated, immobilized and recycled corncob pieces (after the eighth production cycle) were dried in an oven at 50 °C overnight and analysed with scanning electron microscopy (JSM-6380 A, JEOL, Peabody, MA, USA).

### 2.8. Collection of Bagasse and Its Pretreatment

Sugarcane bagasse was collected from a local fruit-juice shop in Karachi, Pakistan. The material was washed and dried at 60 °C for 48 h. The dried material was ground to 300 µ pore size and stored.

### 2.9. Saccharification of Bagasse by Crude Xylanase

Hydrolysis of commercial xylan and sugarcane bagasse through a crude xylanase preparation of S. cerevisiae MK-157 was carried out. A substrate (1%) was added to 100 mL of 50 mM sodium citrate buffer (pH 4.8), which also contained 0.2% (*w*/*v*) Na-azide and 10 units of the enzyme. The mixture was incubated at 37 °C for 52 h with agitation (100 rpm). Aliquots were withdrawn at different time intervals (1 h, 24 h, 48 h and 52 h) and centrifuged at 7000 rpm for 15 min. The supernatant was collected and assayed to quantify the amount of reducing sugars.

### 2.10. Reducing Sugar Assay

Supernatant (25 µL) was added to 25 µL of 50 mM sodium citrate buffer (pH 4.8). Afterwards, 150 μL of DNS reagent was added and the mixture was boiled for 5 min. After boiling, distilled water (720 μL) was added to the solution and the absorbance at 540 nm was recorded. Reducing sugars were calculated by comparing the OD_540_ of the sample with the standard solutions of xylose [19].

### 2.11. Statistical Analysis

All the experiments were performed in triplicate. The average and standard deviation were calculated using the software Origin 8. The results are represented as mean values with the non-significant standard deviation.

## 3. Results and Discussion

Exploring biodiversity in the search for new biocatalysts produced by indigenous microorganisms may permit the development of biocatalysts at the commercial level. Previously, various microbial strains, including *Penicillium* sp., *Aspergillus niger*, *Bacillus* sp., *Thermobacillus xylanilyticus*, *Aspergillus oryzae* and *Aspergillus foetidus*, have been reported to produce xylanase [20]. In spite of the availability of high-throughput genetic engineering techniques, the isolation of novel indigenous strains with the ability to overproduce metabolites of commercial importance has remained at the core of industrial microbiology research. Interestingly, only one strain of *S. cerevisiae*, labelled SCPW 17 and isolated from a soil sample from a mushroom farm in the Yala Local Government Area of Cross River State, Nigeria, has been reported as suitable for xylanase production [21]. Otherwise, being an ideal eukaryotic host for genetic manipulation, heterologous xylanase production from *S. cerevisiae* has been widely reported [22]. Therefore, this report follows the earlier report about SCPW 17 regarding xylanase production. The strain MK-157 differs from the SCPW 17 strain owing to the fact that its cellulolytic [9] and pectinolytic [8] potential has been previously reported. Nonetheless, extensive genetic characterization needs to be undertaken to elucidate the polysaccharide degradation potential completely. In this study, *S. cerevisiae* MK-157 was used for xylanase production. The composition of the production medium plays a vital role in the regulation of an organism’s growth and the production of metabolites. In this study, three different media were screened to investigate the production of xylanase. *S. cerevisiae* MK-157 produced the maximum xylanase (0.48 IU mL^−1^) when cultivated in MSM medium and did not produce an appreciable titer in the other two media (Table 1). It was found that 0.38 IU mL^−1^ and 0.22 IU mL^−1^ xylanase were produced in YEB and PBM, respectively. In contrast, Qinnghe et al. [23] reported that ligninolytic enzyme activity was stimulated in a nitrogen-rich medium, as the nitrogen source had a dramatic effect on the production of xylanase. A nitrogen source can significantly affect the pH of a medium during the course of fermentation; hence, enzyme activity and stability are affected if the nitrogen source is not optimal [24]. Since only 0.48 IU mL^−1^ of xylanase was obtained initially, an immobilization method was adopted to enhance the xylanase titers. In place of genetic manipulation, immobilization of strains has become widely accepted as a safe process that can effectively reduce the cost of production. Therefore, corncob was here utilized as an immobilization matrix. Immobilized yeast cells on corncob produced 4.97 IU mL^−1^ xylanase, indicating an improvement of more than tenfold in xylanase titers compared to the initial experiments. Hence, the objective of the immobilization—to increase xylanase yield—was achieved. Various matrices have been explored for yeasts, including a poly-l-lactic acid (PLLA) microtube array membrane for *Kluyveromyces marxianus* [25], a chamotte carrier for *Saccharomyces pastorianus* 680 [26], cashew apple bagasse for *S. cerevisiae* [27] and spent grains for *Kluyveromyces marxianus* CCT 3172 [28]. However, corncob—as an immobilization matrix for *S. cerevisiae* in general and for xylanase production in particular—has not been studied until now. In addition to its wide availability and low cost, corncob also possesses various desirable attributes that make it an ideal immobilization matrix, including mechanical stability and a porous nature [12]. Alkali pretreatment of corncob was performed in order to make the matrix more porous. Sodium hydroxide degrades the lignin component present within the cell wall of corncob. The treatment makes the matrix more porous and the cells can easily embed within the cavities of the corncob. Hence, more cells are immobilized, leading to enhanced production of xylanase. Weight analysis of the matrix after immobilization showed that there was an average increase of 44 ± 3 mg in the weight of the corncob. The increase in the weight of the corncob after immobilization was indicative of the adherence and growth of the cells on the matrix.

**Figure 3:**
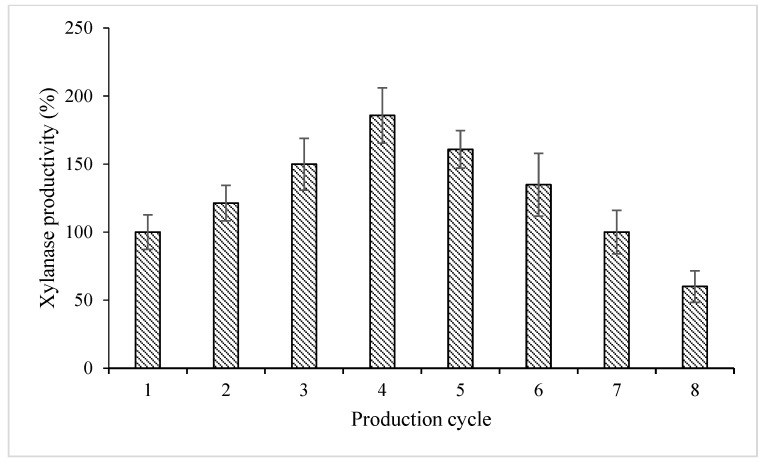
Xylanase production from *S. cerevisiae* MK-157 in different production cycles. Xylanase activity is expressed as percentages, and activity after the first production cycle was taken as 100% (100% enzyme activity = 4.97 IU mL^−1^).

Immobilized cells have various advantages over free cells; for instance, immobilized cells are shielded from environmental insults and can be applied in immobilized cell reactors in a continuous manner. *S. cerevisiae* MK-157 produced 4.97 IU mL^−1^ in the first production cycle (Table 2). Various moulds, including *Aspergillus flavus*, *A. terrus*, *Penicillium atrovenetum* and *P. expansum*, were found to produce only <3.7 IU mL^−1^ xylanase [29], which was less than the titers obtained in this study. Moulds have been reported to produce multi-enzyme outcomes that result in additional steps in the downstream production of xylanase. In contrast, yeast produces fewer extracellular plant cell wall-degrading enzymes during a shorter cultivation period and requires simple nutrients [30]; hence, it is preferred over moulds. The recycling of immobilized MK-157 cells showed 21.32, 49.89, 85.71, 60.76 and 34.82% increments in xylanase production in the second, third, fourth, fifth and sixth production cycles, respectively (Figure 3). It has been reported that the confinement of cells to specific regions preserves biocatalytic activity and renders the cells recyclable [31].

Various yield parameters were investigated to compare the efficiencies of different processes. The volumetric productivity parameter provides the yield of a process in relation to the batch volume and time duration and, hence, is an effective measure of a bioprocess. Bioprocesses with high volumetric productivity are easy to scale up in a cost-effective manner [32]. The volumetric productivity was calculated to compare the IU mL^−1^ h^−1^ for each of the production cycles [33]. It was found to be 298.2, 361.8, 447, 553.8, 479.4, 402, 297 and 178.8 IU mL^−1^ h^−1^ for the first to eighth production cycles, respectively (Table 3). Xylanase production (9.23 IU mL^−1^) and specific productivity (36.92 IU g^−1^) were the maximum in the fourth cycle and then started to drop subsequently (Table 2 and Table 3). The increased xylanase production and specific productivity can be attributed to the cell invasion to the matrix and subsequent growth. However, matrices do not hold cells for an indefinite period, and consequent leaching of cells from a matrix leads to a decline in production [34]. The degradation and deformities caused by repeated cycles in an acidic medium accompanied by the leaching of cells could be linked to decreased xylanase production and, specifically, its production in the fifth and subsequent cycles. Previously, Qadir et al. [8] reported a decline in pectinase production from immobilized yeast after the third production cycle due to deterioration of the matrix; lysed yeast cells visible in SEM images were attributed to the change in pH or the accumulation of toxic metabolites. However, in this study, the xylanase yield did not decline from 100% (i.e., the production obtained in the first cycle) for up to seven cycles (Figure 3).

**Figure 4:**
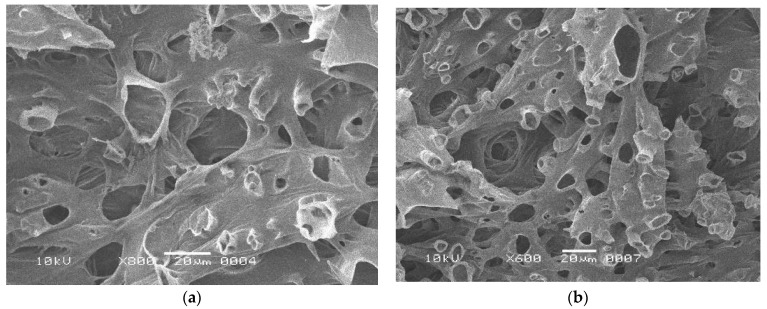

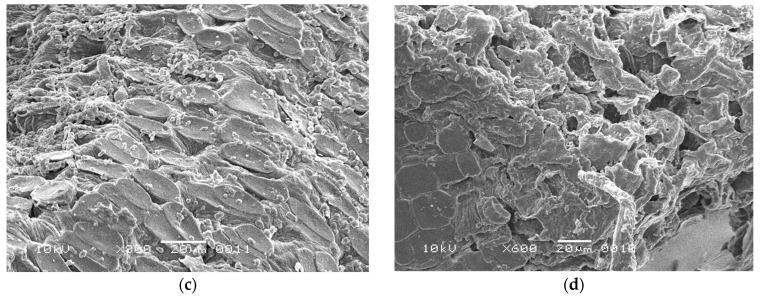
Scanning electron microscopy images of (**a**) corncob prior to alkali pretreatment, (**b**) alkali-pretreated corncob, (**c**) immobilized *S. cerevisiae* MK-157 on treated corncob and (**d**) recycled corncob.

Structural analysis of corncob after alkali pretreatment and immobilization was undertaken using SEM (Figure 4). The variable structure of the corncob was itself advantageous to the process of immobilization, as a larger surface was available for the attachment of the cells (Figure 4a) [35]. However, the matrix became more porous after the alkali treatment (Figure 4b), which removed lignin content [36]. Immobilized cells of *S. cerevisiae* MK-157 could clearly be seen in the matrix, which resulted in higher xylanase production as compared to the free cells (Figure 4c). Ndubuisi et al. [37] concluded that corncobs are the best supporting material for immobilization, as, in their study, the cells of *Pichia kudriavzevii* efficiently attached to corncobs with well-stretched and separate morphologies. Better immobilization on alkali-pretreated corncob can be attributed to the structural modifications caused by sodium hydroxide, as revealed by the SEM analysis (Figure 4c) [8]. SEM analysis of recycled corncob showed that its structure was degraded, and yeast cells were leached from the matrix (Figure 4d). This finding was in line with a previous report [38] where degraded corncob structure after the fifth cycle of bioethanol production was observed.

The ability of the crude xylanase preparation from *S. cerevisiae* MK-157 to saccharify sugarcane bagasse was evaluated. Sugarcane bagasse is an abundant lignocellulosic biomass that is generated after extracting juice from sugarcane [39]. The heterogeneity of bagasse requires hemicellulose activity for its degradation [40]. The amount of reducing sugars released (mg mL^−1^) after the hydrolysis of the substrate was taken as a measure of saccharification (Table 4). The data showed that the yeast enzyme yielded greater amounts of reducing sugars in comparison to commercial xylan after the saccharification of sugarcane bagasse at rates of 0.54, 18, 26 and 17.2 mg L^−1^ following 1 h, 24 h, 48 h and 52 h time intervals, respectively. Previously, Mahamud and Gomes [41] reported higher saccharification of alkali-tretaed bagasse using an enzyme preparation of *Trichoderma* sp. compared to *Aspergillus niger*, *Cladosporium* sp. and *Curvularia* sp. In this study, xylanase from *S. cerevisiae* MK-157 was found to be able to saccharify sugarcane bagasse without any prior treatment of the bagasse. However, further studies are required to clarify the mechanism of the yeast enzyme for subsequent utilization in commercial processes.

## 4. Conclusions

*S. cerevisiae* MK-157 produced low titers of xylanase when cultivated in the free-cell state. Mineral salt medium supplemented with xylan appeared to be better than the media containing organic nitrogen and carbon sources. To improve the xylanase titers obtained from MK-157, corncob, an abundantly available agro-waste residue, was successfully used as a solid-state support for xylanase production. Alkali treatment of the matrix resulted in pore formation, as evidenced by scanning electron microscopy. The use of corncob as an immobilization matrix resulted in a ~tenfold increase in the xylanase titers compared to free cells. The immobilized yeast could also be reused in eight sequential fermentation cycles for xylanase production. Furthermore, the crude xylanase preparation efficiently saccharified the untreated bagasse. Based on the observations, it can be suggested that xylanase from *S. cerevisiae* MK-157 can be exploited at the industrial scale for the production of xylanase. 

## Figures and Tables

**Table 1:** Effect of media composition on xylanase produced from *S. cerevisiae* MK-157.

Strain	Xylanase (IU mL^−1^) *		
	Mineral Salt Medium	Yeast Extract Peptone Medium	PeptoneBroth Medium
MK-157	0.48	0.38	0.22

* Insignificant standard deviation.

**Table 2:** Xylanase production from immobilized cells after recycling of matrix for up to eight production cycles.

Production Cycle	Xylanase (IU mL^−1^) *
1	4.97
2	6.03
3	7.45
4	9.23
5	7.99
6	6.7
7	4.95
8	2.98

* Insignificant standard deviation.

**Table 3:** Analysis of cell-free culture supernatant obtained after different numbers of production cycles.

Production Cycle	Analysis *	
	Volumetric Productivity (IU mL^−1^ h^−1^)	Specific Productivity (IU g^−1^ of Xylan)
1	298.2	19.88
2	361.8	24.12
3	447	29.8
4	553.8	36.92
5	479.4	31.96
6	402	26.8
7	297	19.8
8	178.8	11.92

* Insignificant standard deviation.

**Table 4:** Saccharification of bagasse using crude xylanase preparation and amount of reducing sugars released after different time intervals per gram of substrate.

Substrate	Reducing Sugars (mg L^−1^) *			
	1 h	24 h	48 h	52 h
Milled sugarcane bagasse	0.54	18	26	17.2
Xylan	0.36	1.6	2.4	1.8

* Insignificant standard deviation.

## Data Availability

Data associated with this manuscript can be obtained from the corresponding author upon a reasonable request.
